# Personal Care Aides in residential care and adult day centers: differences in training, benefits, and roles

**DOI:** 10.1093/geroni/igab046.3098

**Published:** 2021-12-17

**Authors:** Manisha Sengupta, Farida Ejaz

**Affiliations:** 1 CDC/NCHS, Hyattsville, Maryland, United States; 2 Benjamin Rose Institute on Aging, Cleveland, Ohio, United States

## Abstract

Personal care aides (PCAs), along with other direct care workers, provide the hands-on care, including help with activities of daily living for individuals receiving care in residential care communities (RCC) and adult day services centers (ADSC). Recruitment and retention of such workers is a challenge as low pay, inadequate training, unsatisfactory roles and lack of benefits contribute to turnover. Using data from the 2018 National Study of Long-Term Care Providers, the only nationally representative data about PCAs in RCCs and ADSCs, this study will assess differences in training hours, benefits, and work roles among PCAs in these settings. About 76% of RCCs and 66% of ADSCs employed aides. On average, PCAs received 32 hours and 51 hours of initial training in ADSCs and RCCs, respectively. Results from bivariate analyses (accounting for complex survey design), showed that benefits received by PCAs varied by sector. A higher percentage of PCAs in ADSCs than in RCCs received health insurance for employees (60% vs. 46%), and pension (51% vs. 40%). About 51% of ADSCs and 46% of RCCs reported that PCAs rarely or sometimes attended care plan meetings. Further, 11% of RCCs and 15% of ADSCs reported that aides rarely or sometimes worked with the same care recipient. This overview of PCA activities, training and benefits may provide insights into approaches to improve the retention of PCAs and subsequently the quality of care provided across sectors.

